# A simulation model of neuroprogenitor proliferation dynamics predicts age-related loss of hippocampal neurogenesis but not astrogenesis

**DOI:** 10.1038/s41598-017-16466-3

**Published:** 2017-11-28

**Authors:** Sol Beccari, Jorge Valero, Mirjana Maletic-Savatic, Amanda Sierra

**Affiliations:** 1 Achucarro Basque Center for Neuroscience, Leioa, Bizkaia Spain; 2 University of the Basque Country UPV/EHU, Leioa, Bizkaia Spain; 3 Ikerbasque Foundation, Bilbao, Bizkaia Spain; 4 Jan and Dan Duncan Neurological Research Institute at Texas Children’s Hospital, Baylor College of Medicine, Houston, TX USA

## Abstract

Adult hippocampal neuroprogenitors give rise to both neurons and astrocytes. As neuroprogenitors are lost with increased age, neurogenesis concomitantly decreases. However, the dynamics of neuron and astrocyte generation throughout adulthood has not been systematically examined. Here, we analyzed the hippocampal niche both longitudinally (from 2 h to 30d of cell life) and transversally (from 1 m to 12 m of age) and generated a Marsaglia polar random simulation model to predict newborn cell dynamics. The sharp decrease in newborn neuron production throughout adulthood was largely predicted by the number of proliferating neuroprogenitors at each age. In contrast, newborn astrocyte decay was slower and associated with their increased yield in mature mice. As a result, the niche shifted from neurogenic to neuro/astrogenic with increased age. Our data provide a simple “end-point” model to understand the hippocampal niche changes across adulthood and suggest yet unexplored functions of newborn astrocytes for the aging hippocampal circuitry.

## Introduction

In the adult hippocampus, new neurons are continuously generated throughout life but markedly decline with age in rodents^1–3^ and humans^4,5^. New cells arise from the asymmetric division of radial neural stem cells (NSCs) located in the SGZ (subgranular zone) of the dentate gyrus (DG). Transient amplifying cells (TAPs) subsequently follow a cascade of events that includes proliferation, survival, and differentiation into mature granule neurons^6^. After about 30 days, these cells integrate into the granule cell synaptic circuitry^7^ where they are involved in memory and learning as well as mood control in mice^6,8–11^.

In addition to neurons, newborn astrocytes are also produced in the SGZ neurogenic niche, as initially shown by conventional pulse-and-chase labeling experiments with the thymidine analog BrdU (Bromo-deoxyuridine)^12,13^. More recently, hippocampal NSCs have been observed to directly give rise to astrocytes at both the population and single cell level using targeted transgenic expression of fluorescent reporters^14,15^. Newborn astrocytes participate in the glial scar formed in the hippocampus during epileptic crisis^16^ but their functional contribution to the hippocampal circuitry in physiological conditions has been largely neglected^6^. Furthermore, while the age-related decline of newborn neurons is well reported and associated with a loss of neuroprogenitors (both NSCs and TAPs) or with their reduced mitotic potential (reviewed in^17^), little is known about the generation of newborn astrocytes with increased age.

Here, we set to systematically analyze the differential production of newborn neurons and astrocytes across adulthood. Live imaging of the hippocampal neurogenic niche over the 4-week integration period and through adulthood imposes technical restrictions not currently surmountable. Thus, we resorted to a computational simulation approach to trace the fate of simulated proliferating neuroprogenitors and their offspring across adulthood from 1 month to 12 months of mouse life. We utilized a Marsaglia polar model to generate populations of simulated newborn cells and examine their conversion into neurons or astrocytes from 2 h to 30 days of newborn cell life, based on the data obtained experimentally after the injection of BrdU. The combination of experimentally estimated and computationally simulated data allowed us to illuminate the differential dynamics of newborn neurons and astrocytes throughout adulthood and demonstrate that the age-related newborn neuron decline is due to reduced number of proliferating neuroprogenitors and not counteracted by compensatory mechanisms, such as neuroprogenitor proliferation or newborn cell survival and/or differentiation. In contrast, the yield of newborn astrocytes increased over time and as a result, the niche switched from neurogenic to neuro/astrogenic in mature mice.

## Methods

### Animals and 5-bromo-2′-deoxyuridine (BrdU) injections

All experiments were performed in male C57BL/6JOlaHsd mice purchased from Harlan at 3 weeks of age (after weaning) and maintained in-house until they reached 1, 2, 6 and 12 months. C57 mice were chosen as they are the most widely used strain for transgenic mouse models. The OlaHsd substrain has a deletion in the gene encoding for alpha synuclein, which is involved in the etiopathology of Parkinson’s Disease in the striatum^18^, and may have differences in the aging process compared to other substrains. Nonetheless, it remains widely used and overall is genetically very similar to other C57BL/6 J substrains^19^. Mice were fed a standard diet ad libitum, and housed in a 12 hour light-dark cycle in the animal facility at the University of the Basque Country (UPV/EHU). All procedures were approved by the Ethics Committees of the University of the Basque Country UPV/EHU and were conducted in accordance with European recommendations (European Directive 2010/63/EC). Mice received four intraperitoneal injections of BrdU (150 mg/kg, diluted in 0.1% NaOH, PBS), every 2 h and were sacrificed 2 h, 2d, 4d, 10d, or 30d later.

### Immunofluorescence

Animals were deeply anesthetized and perfused transcardially with PBS followed by 4% paraformaldehyde (PFA) in PBS. After 4 h postfixation in 4% PFA, serial 50 µm thick sagittal sections were collected using systematic-random sampling with a VT 1200 S vibratome (Leica Microsystems GmbH, Wetzlar, Germany) in 6 parallel sets. Immunofluorescence was performed using standard procedures^16^ including a 15 min preincubation with 2 M HCl for 15 min at 37 °C. Primary antibodies were incubated overnight at 4 °C: rat anti-BrdU (1:300; AbD Serotech, Kidlington, UK), rabbit anti-GFAP (1:1000; DakoCytomation, Denmark), rabbit anti-NeuN (1:1000; Abcam Discover, England), and rabbit anti-Ki67 (1:1000; Vector Laboratories, Burlingame, CA, USA). Secondary antibodies were incubated for 2 h at room temperature: RRX-goat anti-rat and Alexa 488-goat anti-rabbit (1:500, Jackson ImmunoResearch, West Grove, PA), and DAPI (5 mg/ml; 1:1000, Sigma). Rinsed slices were mounted on glass slides with Fluorescent Mounting Medium (DakoCytomation, Denmark),).

### BrdU cell quantification

Quantitative analysis of cell populations was performed by using a modified optical fractionator sampling^20^. Total BrdU^+^ cells were counted in 1–6 of the six series in a Zeiss LSM epifluorescence microscopy under a 20X objective. To avoid overestimation, cells whose nuclei were contained in the uppermost focal plane were disregarded. Only cells in the SGZ or the granule cell layer were counted, whereas cells in the hilus or the molecular layer were excluded. Individual BrdU^+^ cells were identified based on DAPI nuclear staining. A 40x objective was used to unequivocally identify single BrdU^+^ nuclei in highly dense cell clusters (Fig. S1). Total BrdU^+^ cells counted were extrapolated to the whole or the septal hippocampus (spanning from −1 mm to −2.5 mm in the AP axes, from Bregma; approximately six slices in each of the six series). Experiments involving marker expression of BrdU^+^ cells (minimum of 50 cells per animal, randomly selected) were performed in z-stacks obtained in a Leica SP8 laser scanning microscope using a 63X oil-immersion objective and a z-step of 0.70 µm. Brightness and contrast were adjusted equally for the entire image in each channel (RGB format) using Adobe Photoshop 7.0 (Adobe Systems Incorporated, San Jose, CA). Raw data of cell counting is shown in Tables S1–S5.

### Statistical analysis

The normality of each of the experimentally quantified populations of BrdU^+^ cells along the time course (from 2 h to 30d) and across ages (from 1 m to 12 m), as well as those expressing NeuN and GFAP was assessed using the Shapiro Wilk test using SigmaPlot 11.0 (Systat Software, San Jose, CA). Only two of the 24 populations analyzed (BrdU^+^ cells at 10d in 2 m mice, and BrdU^+^ cells at 30d in 12 m mice) did not follow a normal distribution. The effect of age and time of injection in was analyzed by two-way analysis of variance (ANOVA), followed by posthoc analysis with Holm-Sidak’s test. Neuronal and astrocytic yield values were not normally distributed, thus they were analyzed by Kruskal-Wallis analysis, followed by posthoc analysis with Tukey’s test. Only p < 0.05 is reported to be significant. Data in graphs is expressed as mean ± SEM (standard error of the mean). N, mean, and standard deviation values for experimentally determined and Marsaglia simulated data (see below) are shown in Table S6. Data modeling was performed using GraphPad Prism 5 (GraphPad Software, Incl, San Diego, CA) and the fitting of different models was compared in GraphPad using Akaike’s information criterion with correction for finite sample sizes (AICc)^21^ to determine the optimal fitting.

### Generation and analysis of pseudorandom simulated data

Marsaglia polar method^22^ was implemented to generate pseudorandom and normally distributed populations of BrdU^+^ cells and their progeny with mean and standard deviation corresponding to those experimentally determined in each age. Groups of nested pseudorandom data were generated following these biological-related restrictions: 2 h ≤ 2d ≥ 4d ≥ 10d ≥ 30d ≥ (GFAP^+^  + NeuN^+^). Two possible strategies were compared to satisfy that the sum of GFAP^+^ and NeuN^+^ newborn cells was less than the total amount of newborn cells found at 30d [(GFAP^+^  + NeuN^+^) ≤ 30d]: randomly determine the percentage of GFAP^+^ cells and condition the proportion of NeuN^+^ to this value (GFAP-locked), or vice versa (NeuN-locked). We chose the GFAP-locked strategy, as it rendered a smaller deviation of the simulated populations from the experimentally estimated populations (4.7% vs. 7.6%, respectively). Nonetheless, the main findings reported here are also reached using the NeuN-locked strategy (Supplementary Text).

### Data availability

The datasets generated and/or analyzed in this study are included in the Supplementary Text (Tables S1–S6).

## Results

### The newborn cell dynamics in the hippocampal niche is similar throughout adulthood

**Figure 1:**
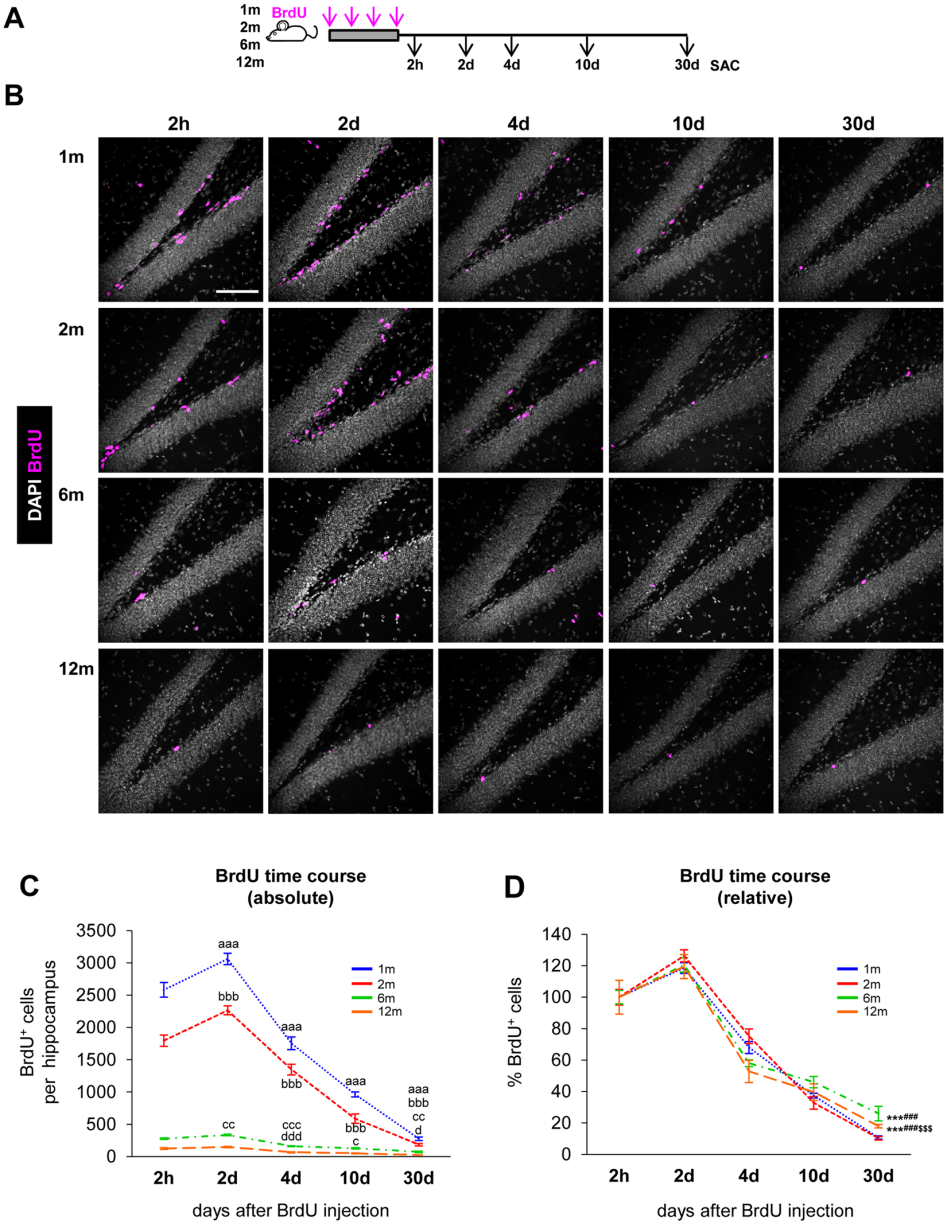
Age-related decline in hippocampal neurogenesis. (**A**) Experimental design used to analyze the neurogenic cascade in young (1 and 2 m) and mature (6 and 12 m) mice. Mice received an injection of BrdU every 2 h for 6 h (4x total) and were sacrificed (SAC) at different time points after the last injection. (**B**) Representative confocal z-stacks of the dentate gyrus (DG) of 1, 2, 6 and 12 m mice at 2 h, 2d, 4d, 10d, and 30d after the BrdU injections (magenta). DAPI staining indicates cell nuclei and the outline of the DG. Scale bar = 100 µm; z = 16.5 µm. (**C)** Absolute number of BrdU^+^ cells per hippocampus along the BrdU time course. N = 5 for 1mo and 2mo mice at 2 h, 2d, 4d, 10d, 30d; N = 4 for 6mo mice at 2 h, 2d, 4d, 10d, 30d; N = 7 for 12mo mice at 2 h, 2d, 4d and 10d, and N = 10 for 12mo mice at 30d. 2-way ANOVA (time after injection × age) showed a significant interaction between the two variables F(12, 88) = 122.982, p < 0.001. Thus 1-way ANOVA was used to analyze statistical differences due to the time after BrdU injection at each age: F(4, 20) = 199.587, p < 0.001 for 1 m; F(4,20) = 145.638, p < 0.001 for 2 m; F(4,15) = 97.491, p < 0.001 for 6 m; and F(4,33) = 43.665, p < 0.001 for 12 m. Holm-Sidak was used as a posthoc test. a, b, c and d represent significance compared to the prior time point for 1 m, 2 m, 6 m and 12 m mice, respectively. One symbol represents p < 0.05, two: p < 0.01, and three: p < 0.001. The number of BrdU^+^ cells in the septal hippocampus is shown in Fig. S2A. Raw data is shown in Table S1. (**D**) Percentage of BrdU^+^ cells per hippocampus. Absolute cell count was normalized to the number of BrdU^+^ cells found at 2 h. N as in Fig. 1C. 2-way ANOVA (time after injection × age) showed no significant interaction between the two variables: F(12, 88) = 1.463, p = 0.154; no significant effect of age: F(3,88) = 0.553, p = 0.647; and a significant effect of the time after injection: F(4,88) = 237.431, p < 0.001. While no overall effect of age was found, we analyzed statistical differences due to the age at each time point after the injection using 1-way ANOVA: F(3,17) = 0.33, p = 0.814 at 2d; F(3,17) = 3.329, P = 0.045 at 4d (although posthoc analysis by Holm-Sidak did not reveal any significant differences); F(3,17) = 1.364, p = 0.287 at 10d; F(3,20) = 91.088, p < 0.001 at 30d. Holm-Sidak was used as a posthoc test. The symbols indicate p < 0.01 between 2 m vs. 12 m; and ^*, #, $^ represent p < 0.001 between 1 and 2 m vs. 6 m, 1 and 2 m vs. 12 m, and 6 m vs. 12 m, respectively. The percentage of BrdU^+^ cells in the septal hippocampus is shown in Fig. S2B. The percentage of BrdU^+^ cells expressing Ki67 at 2d is shown in Fig. S2C,D. N = 10 at 1 m, 2 m; N = 8 at 6 m; N = 14 at 12 m. Raw data is shown in Table S4. Points represent mean ± SEM.

The decay of proliferating neuroprogenitors across age as well as the decay of newborn neurons along the BrdU time course at particular ages are well documented^1–3,14,23,24^. Here we have performed a systematical analysis of the decay of both newborn neurons and newborn astrocytes: i) longitudinally (from 2 h to 30d of cell life), and ii) transversally (at 1 m to 12 m of age). In both experimental designs, we used a semicumulative BrdU paradigm, in which BrdU was injected at 150 mg/kg every 2 h for 6 h^16^ (4x BrdU; Fig. 1A). The saturating dose of BrdU^24^ prevents dilution of the BrdU label over the time period investigated^25^. In addition, a repeated injections paradigm increases the number of labeled cells and maximizes the probability of observing BrdU^+^ cells in older animals, particularly at later stages of the neurogenic cascade.

The number of BrdU^+^ cells in the granular cell layer (GCL) and subgranular zone (SGZ) of the hippocampus declined with increased age and this was evident at all time points studied (Fig. 1B,C), in agreement with previous reports^1–3,14,23,24^. We found very little evidence of BrdU^+^ cells in the hilus or molecular layer that could hinder the interpretation of our results. At all ages studied (1–12 months), the maximum number of BrdU^+^ cells in the whole hippocampus was detected at 2d (Fig. 1B), reflecting a period of net cell expansion between 2 h and 2d. The same pattern was observed in the septal portion of the hippocampus, in spite of receiving different innervation compared to the temporal portion^26^ (Fig. S2A). The peak of cell production at 2d was to some extent smaller compared to other studies that used a single BrdU injection paradigm (1x BrdU)^13,14,24,25^. This peak is not detected in longer cumulative labeling paradigms (8x BrdU, injected every 3 h for 24 h)^25^, suggesting that in multiple BrdU injection paradigms the overlap between the cell cycle progression and survival of the first labeled cohort as well as the continued labeling of subsequent cohorts of proliferating cells complicates the interpretation of the proliferation peak.

After the 2d peak, the number of BrdU^+^ cells significantly declined up to 30d at all ages examined (Fig. 1C). Further, we found no major differences in the overall BrdU^+^ time course when the number of BrdU^+^ cells was normalized to the number at 2 h at each age, either in the whole or the septal hippocampus (Fig. 1D; Fig. S2B). Thus, these data suggest that the dynamics of newborn cells across the time course of 30 days largely depends on the initial number of proliferating neuroprogenitors labeled with BrdU at 2 h. To further test this observation, we analyzed in more detail each step of the neurogenic cascade throughout adulthood, focusing on proliferation, survival, and differentiation.

We first examined the period of increased net proliferation between 2 h and 2d. This peak results from the combination of cell cycle completion, further divisions, and ongoing apoptosis^25^. We found no significant differences in the relative increase of newborn cells from 2 h to 2d across ages (Fig. 1D), suggesting that the net increment of cells (proliferation minus apoptosis) is maintained through adulthood. To further delve into the proliferation of 2d cells, we analyzed the expression of the proliferation marker Ki67, expressed in cells during all active phases of the cell cycle^27^. We found that 2d BrdU^+^ cells were mitotically active at similar rates from 1 m to 12 m (Fig. S2C,D). While a more detailed analysis of the cell cycle duration of the different neuroprogenitor populations may reveal age-induced changes, our data show similar net increments at 2d and similar Ki67 expression, and are consistent with no major differences in the proliferation capacity of the neuroprogenitor population up to 12 m.

### The critical periods of newborn cell survival are maintained across adulthood

**Figure 2:**
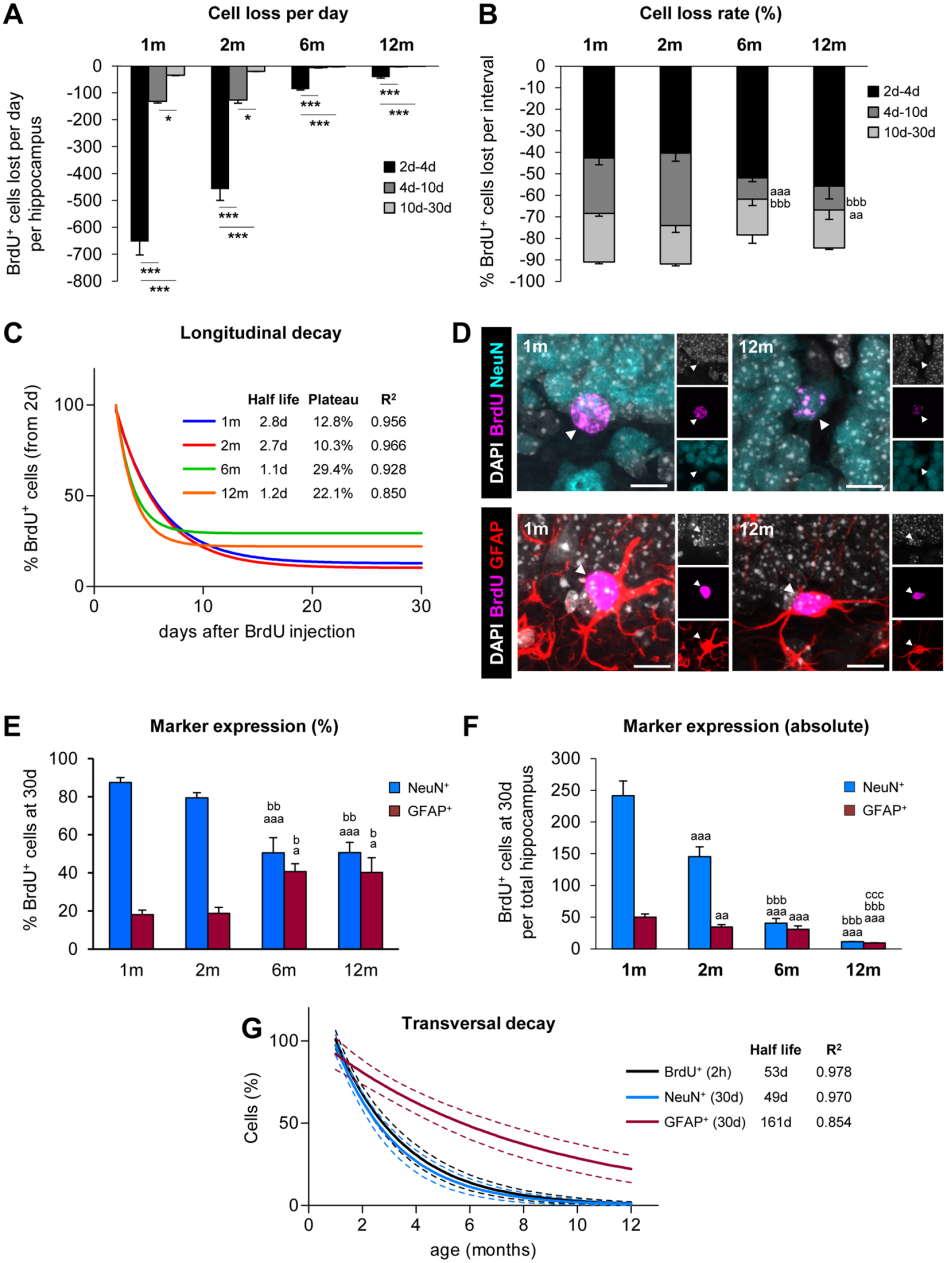
Longitudinal and transversal decays of newborn cell populations. (**A**) Loss of BrdU^+^ cells per day, calculated as the total number of BrdU^+^ cells lost in the hippocampus during the periods 2d–4d, 4d–10d, and 10d–30d. N per group as in Fig. 1C. The effect of age in the loss of BrdU^+^ cells per period was analyzed using 1-way ANOVA: F(2,12) = 134.198, p < 0.001 at 1 m; F(2, 12) = 83.114, p < 0.001 at 2 m; F(2,9) = 553.774, p < 0.001 at 6 m; F(2, 21) = 94.635, p < 0.001. Holm-Sidak was used as a posthoc test.*, ** and *** indicate p < 0.05, p < 0.01 and p < 0.001 between periods for each age, respectively. (**B**) Cell loss rate of BrdU^+^ cells, calculated as the percentage of BrdU^+^ cells lost from the number of BrdU^+^ cells at 2d at each interval in the hippocampus. N per group as in Fig. 1C. The effect of the period in the loss of BrdU^+^ cells at each age was analyzed using 1-way ANOVA: F(3,17) = 2.593, p = 0.086 at 2d–4d; F(3,17) = 10.098, p < 0.001 at 4d–10d; F(3,20) = 2.66, p = 0.076 at 10–30d. Holm-Sidak was used as a posthoc test. a, b, and c represent significance respect to 1 m, 2 m, and 6 m, respectively. Two symbols are used for p < 0.01 and three for p < 0.001. Bars represent mean ± SEM. (**C**) Longitudinal decay of BrdU^+^ cells over the time course (2d–30d), calculated as an exponential curve with plateau. Fitting curve, cell half-life, survival plateau (%), and R^2^ are indicated (further data is shown in Table S7). The longitudinal decay of 1 m mice in 1x BrdU, 4x BrdU, and 8x BrdU paradigms is shown in Fig. S2E. (**D**) Representative confocal z-stacks of the DG of 1 and 12 m mice at 30d after the BrdU injections. BrdU^+^ cells (magenta) were co-labeled with the mature neuronal marker NeuN (in cyan) or the astrocyte marker GFAP (in red). DAPI staining indicated cell nuclei (in white). Scale bars = 10 µm; z = 14 µm. (**E**) Expression of NeuN and GFAP in the BrdU^+^ cells at 30d (in %). N = 4 at 1 m and 2 m, N = 6 at 6 m, and N = 5 at 12 m for % NeuN^+^ cells. N = 9 at 1 m and 2 m, N = 10 at 6 m, and N = 20 at 12 m for % GFAP^+^ cells.1-way ANOVA analysis was F(3,15) = 10.913, p < 0.001 for % NeuN^+^ cells and F(3,44) = 4.18, p = 0.011 for % GFAP^+^ cells. Holm-Sidak was used as a posthoc test. a, b, and c represent significance respect to 1 m, 2 m, and 6 m, respectively. Two symbols are used to represent p < 0.01 and three for p < 0.001. Bars represent mean ± SEM. Raw data is shown in Tables S2 and S3. (**F**) Absolute number of newborn neurons (BrdU^+^, NeuN^+^) and astrocytes (BrdU^+^, GFAP^+^) at 30d in the hippocampus. N = 5 at 1 m and 2 m, N = 4 at 6 m, and N = 15 at 12 m for % NeuN^+^ BrdU^+^ cells; N = 5 at 1 m and 2 m, N = 4 at 6 m, and N = 15 at 12 m for GFAP^+^, BrdU^+^ cells.1-way ANOVA analysis was F (3,25) = 115.154, p < 0.001 for NeuN^+^, BrdU^+^ cells and F(3,25) = 53.589, p < 0.001 or GFAP^+^, BrdU^+^ cells. a, b, and c represent significance respect to 1 m, 2 m, and 6 m, respectively. Two symbols are used to represent p < 0.01 and three for p < 0.001. Bars represent mean ± SEM. (**G**) Transversal decay of BrdU^+^ (2 h), BrdU^+^ NeuN^+^ (30d) and BrdU^+^ GFAP^+^ (30d), is best fitted with an exponential curve. Fitting curve, cell half-life, and R^2^ are indicated (further data is shown in Table S7). The transversal decay of human neuroblasts labeled with doublecortin is shown in Fig. S2F,G. N per group as in Fig. 2F.

After the period of net proliferation, the number of newborn cells declines due to ongoing apoptosis^25^. In physiological conditions and up to 12 m, apoptotic newborn cells are rapidly cleared by unchallenged resident microglia and, as a result, they are very difficult to quantify, particularly in older mice^25^. Instead of quantifying newborn cell death, here we have focused on analyzing their total numbers as a measure of net survival, which reflects the difference between ongoing proliferation and apoptosis, in three periods: 2–4d, 4–10d, and 10–30d. At all ages, the highest rate of cell loss per day occurred in the first 2–4d (Fig. 2A), indicating that the main critical period of survival was maintained. Indeed, 40–56% of the newborn cells were lost in the 2–4d period from 1–12 m (Fig. 2B). Mature mice (6, 12 m) had a small but significant decrease in the cell loss rate in the 4–10d period (26–34% in 1–2 m vs. 10–11% in 6–12 m) (Fig. 2B), resulting in an overall increased net survival at 30d compared to young mice (Fig. 1D).

To further understand the longitudinal decay of the BrdU^+^ labeled cells at each age after the 2d peak, we mathematically modeled their behavior over time. The best fitting was obtained when each age population was adjusted to an exponential decay curve with plateau (Fig. 2C; Table S7). The BrdU^+^ cell decay from 2d to 30d was remarkably similar in young (1–2 m) mice, with a half-life of 2.7–2.8d and a survival plateau reached at 10.3–12.8% (of the 2d peak). Similar parameters were obtained in a set of independent data previously published and obtained from 1 m mice using either 1x BrdU or 8x BrdU paradigms^25^, validating the results obtained here and the possibility of BrdU dilution (Fig. S2E; Table S7). In mature mice (6–12 m), in contrast, BrdU^+^ cell half-life was shorter (1.1d) but more cells survived and the survival plateau was reached at 22.1–29.4% (of the 2d peak).

### Neuron and astrocyte productions are inversely regulated across adulthood

Finally, we examined whether increased age would influence differentiation of newborn cells into neurons vs. astrocytes by analyzing the expression of neuronal NeuN or astrocytic GFAP in BrdU^+^ cells at 30d. Astrocytes were also identified based on their ramified (i.e., non-radial) morphology, to disregard the quantification of potential label-retaining radial NSCs. Because both detecting antibodies were made in rabbit and used in adjacent sections, their combined number only roughly added to 100%. Nonetheless, it is possible that BrdU labels not only newborn cells derived from radial NSCs but also other cell populations such as microglia, although they proliferate at very low levels^28^. The percentage of newborn neurons (NeuN^+^, BrdU^+^) decreased significantly from 6 m onwards (51–56%) compared to 1–2 m (79–87%). In contrast, the percentage of newborn astrocytes (GFAP^+^, BrdU^+^) increased significantly at 6–12 m (40–44%) compared to 1–2 m (18–19%; Fig. 2D,E). The number of newborn neurons decayed continuously from 1 m to 12 m, in a pattern reminiscent to that of proliferating neuroprogenitors (BrdU^+^ cells at 2 h) (Fig. 1C). In contrast, the number of newborn astrocytes remained largely constant through adulthood and decreased at a very slow rate (Fig. 2F).

Next, we compared the dynamics of the three populations (proliferating neuroprogenitors, newborn neurons, and newborn astrocytes) and modeled their transversal decay across adulthood. The optimal fitting was obtained using exponential decay curves (Fig. 2G; Table S7) and the decay occurred at very different rates. The population of proliferating neuroprogenitors and newborn neurons decayed throughout adulthood at a high rate, leading to a population half-life of 53 and 49d, respectively.

Intrigued by the fast decay of newborn neurons, we performed a similar analysis of a sample of doublecortin-labeled neuroblasts from human tissue, previously published^5^. The optimal fitting was obtained when the samples were split into two populations, younger and older than 2 years-of-age (y). Importantly, the 2 y splitting point coincides with a previously identified maturation benchmark of the human brain, at 2–3 y when it reaches 90–95% of adult brain weight^29^. Similarly, the human hippocampal volume increases sharply up to 2 y and more slowly afterwards^30^. We found that in infants under 2 y, the density of neuroblast decayed exponentially with a half-life of 191d. The exponential decay of neuroblast density and the roughly linear increase in hippocampal volume from birth up to 2 y suggests that the total number of neuroblasts decays more exponentially with increased time. After the 2 y benchmark and through adulthood the hippocampal volume is fairly constant and starts to decrease only in advanced age (over 70 y) in healthy people^31^. We found that in children over 2 y and up to 100 y the loss of neuroblast density slowed down and they decayed with a half-life of 25 y (Fig. S2F,G; Table S7).

In contrast, the population of newborn astrocytes in the mouse hippocampus decayed at a slower rate and had a relatively long half-life of 161d (Fig. 2G). These data suggested a differential production of neurons and astrocytes across adulthood and prompted us to analyze the neurogenic and astrogenic yield of the primary proliferating neuroprogenitors.

### The age-related decrease in neurogenesis is predicted by the proliferation of neuroprogenitors

**Figure 3:**
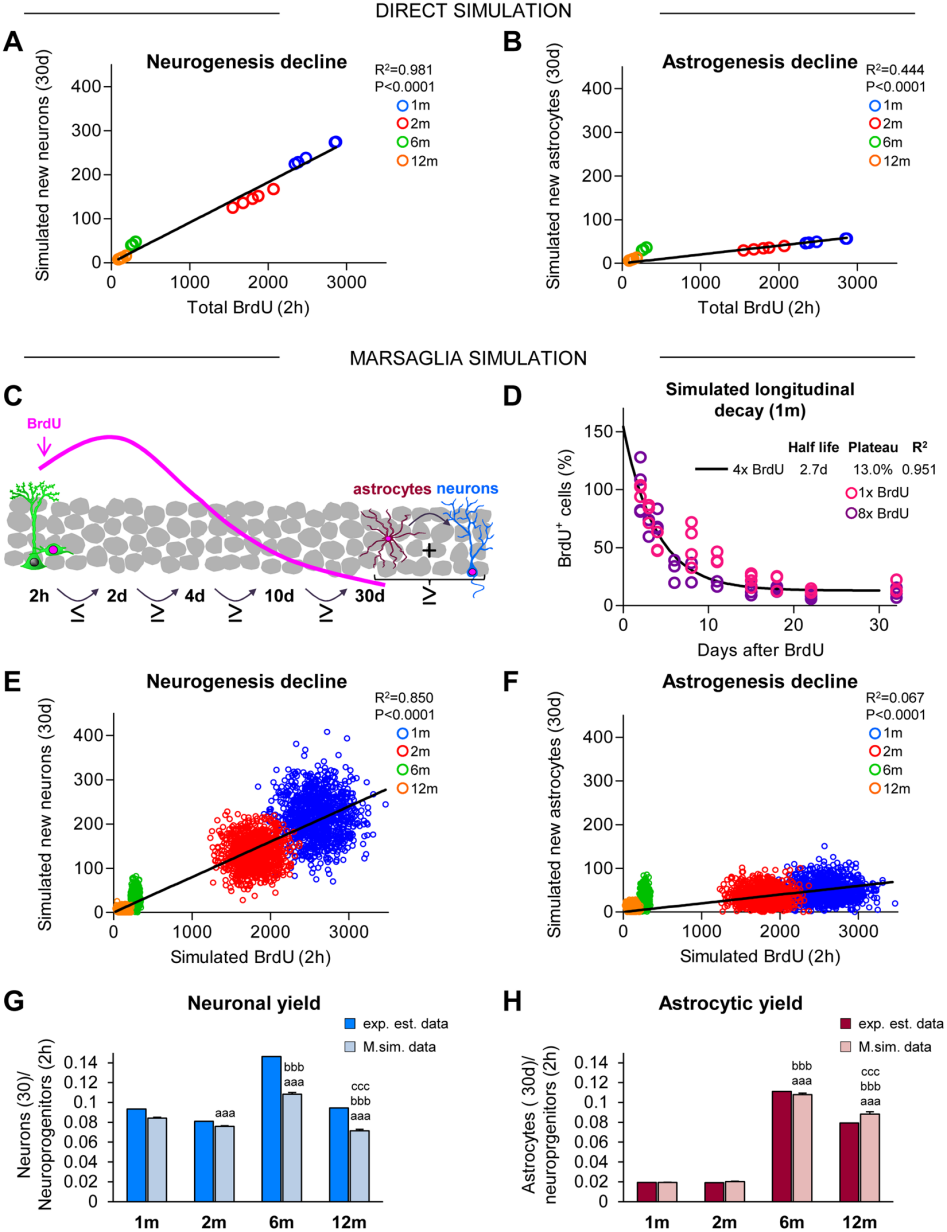
Neurogenesis but not astrogenesis decline is due to the disappearance of proliferating neuroprogenitors. (**A**,**B**) Linear regression of the number of newborn neurons (**A**) and astrocytes (**B**) at 30d generated from proliferating progenitors at 2 h across adulthood. Estimated number of BrdU^+^ cells at 2 h (Fig. 1C) were correlated with the simulated number of BrdU^+^ NeuN^+^ or BrdU^+^ GFAP^+^ cells at 30d, calculated from the estimated survival and differentiation rates (Figs 1D and 2E, respectively). Each dot represents an individual value. The linear regression curve, R^2^, and p-value are shown (further data is shown in Table S8). N per group as in Fig. 2F. (**C**) Cartoon representing the Marsaglia simulation model restrictions based on Figs 1 and 2. In the model, the number of simulated BrdU^+^ cells at 2d must be equal or larger than at 2 h. After 2d, each time point should have the same or fewer BrdU^+^ cells. At the end of the 30d period, the sum of newborn astrocytes and neurons should be equal or smaller than the total BrdU^+^ cells at 30d. A comparison between the experimentally estimated and the Marsaglia simulated data is shown in Table S6. (**D**) Longitudinal decay of 1 m simulated BrdU^+^ cells over the time course (2d–30d), calculated as an exponential curve with plateau. Fitting curve, cell half-life, survival plateau (%), and R^2^ are indicated (further data is shown in Table S7). Individual dots represent two independent sets of BrdU data from single (1x BrdU) and cumulative (8x BrdU) injection paradigms^25^. N = 3 for 2dp, 3dp, 4dp, 11dp, 18dp and 32dp and N = 4dp, 8dp, 15dp, 22dp in 1x BrdU; N = 3 for 6dp, 8dp, 11dp, 15dp, 22dp, and 32dp and N = 4 for 2dp, 4dp, 18dp for 8x BrdU. (**E**,**F**) Linear regression analysis of the simulated number of newborn neurons (**E**) and astrocytes (**F**) at 30d, generated from the proliferating progenitors at 2 h across adulthood using the Marsaglia polar simulation model. Pseudorandom simulated numbers of BrdU^+^ cells at 2 h and BrdU^+^ NeuN^+^ or BrdU^+^ GFAP^+^ cells at 30d were obtained using the mean and standard deviation values (Figs 1C,D and 2E, Table S6). 1,000 pseudorandom cells per age group for each population were generated. Each dot represents an individual value. The linear regression curve, R^2^, and p-value are shown (further data is shown in Table S8). (**G**,**H**) Neuronal (**G**) and astrocytic (**H**) yield of proliferating neuroprecursors derived from the experimentally estimated and the Marsaglia simulated data. Kruskal-Wallis analysis was H(3) = 425.9, p < 0.001 for neuronal yield, and H(3) = 2367, p < 0.001 for astrocitic yield. a, b, and c represent significance compared to 1 m, 2 m, and 6 m, respectively (p < 0.05) for Marsaglia simulated data. Two symbols are used to represent p < 0.01 and three for p < 0.001. Bars represent mean ± SEM. Further data is shown in Table S11.

Current methodology does not allow following the fate of newborn cells in the hippocampus in real time, but only in fixed tissue at different time points. Therefore, to examine the relationship between proliferating neuroprogenitors and their offspring, we turned to mathematical modeling, commonly used to estimate parameters experimentally difficult to derive^32,33^. From the proliferating neuroprogenitors at 2 h, we extracted the mean survival and differentiation rates experimentally estimated at 30d to estimate the number of simulated neurons and astrocytes produced at each age. We found very significant linear correlation between the number of proliferating neuroprogenitors and simulated newborn neurons from 1 m to 12 m (R^2^ = 0.981). In contrast, the number of simulated newborn astrocytes was poorly predicted by the number of proliferating neuroprogenitors across adulthood (R^2^ = 0.444)(Fig. 3A,B; Table S8). However, this direct simulation was based only on the mean values and did not take into account the biological variability of the populations.

Thus, we generated a pseudorandom simulation model using a Marsaglia polar method that took into account both the mean and standard deviation of the survival and differentiation rates estimated experimentally. In this discrete (i.e., non-continuous) simulation model we imposed a few biological restrictions based on the observed data (Figs 1 and 2): i) that the maximum number of BrdU^+^ cells needs to be reached at 2d; ii) that the number of BrdU+ cells continuously decays afterwards; and iii) that at 30d the total number of BrdU+ cells is the sum of neurons and astrocytes (Fig. 3C). With these biologically-imposed restrictions, two models are possible: simulate the number of newborn astrocytes and calculate the number of newborn neurons as the total BrdU+ cells at 30d minus the number of newborn astrocytes (GFAP-locked model) or vice versa, simulate the number of newborn neurons and calculate the number of newborn astrocytes (NeuN-locked model; Tables S9–S11). Here we present the results for the GFAP-locked model, which showed a smaller deviation from the experimentally estimated data compared to the NeuN-locked model (4.7% vs. 7.6%, respectively; Table S6). Then, we generated pseudorandom populations of 1000 BrdU^+^ labeled neuroprogenitors and their offspring at 2, 4, 10, and 30d for each age.

We validated the Marsaglia simulation model obtained from our experimentally estimated data (4x BrdU) and compared it against an independent set of data, previously obtained in 1 m mice using 1x BrdU and 8x BrdU paradigms at different time points (2, 3, 4, 8, 11, 15, 18, 22 and 32d)^25^. First, we generated the simulated longitudinal decay curve for 1 m mice (Fig. 3D; Table S7; R^2^ = 0.951), which indeed produced a similar cell half-life and survival plateau as the fitting curve obtained with the experimentally estimated data (Fig. 2C). Next, we compared the simulated 4x BrdU curve to the experimental 1x and 8x BrdU longitudinal decays by calculating the difference between the mean value of BrdU^+^ cell number for each time point and the value predicted from the simulated longitudinal decay curve (Fig. 3D). Overall, we found no significant differences between our Marsaglia simulation model and the independent set of samples obtained with 1x BrdU (p = 0.1124) and 8x BrdU (p = 0.0822) paradigms or with the experimentally estimated 4x BrdU (p = 0.9448), validating the use of this model to analyze BrdU^+^ newborn cell dynamics.

We then used the Marsaglia simulated data to examine the relationship between proliferating neuroprogenitors and their progeny. We found a strong linear correlation between the number of proliferating neuroprogenitors (at 2d) and the number of newborn neurons produced from them and detected 30d later, at all ages examined (R^2^ = 0.850) (Fig. 3E; Table S8). In contrast, the number of astrocytes generated at different ages was poorly explained by the number of initial proliferating neuroprogenitors (R^2^ = 0.067). Furthermore, in 6–12 m mice, the number of simulated astrocytes was largely above the fitting curve of the astrogenesis decline (Fig. 3F; Table S8), suggesting a differential production of astrocytes from neuroprogenitors across adulthood.

To further confirm this difference in the production of astrocytes across ages, we calculated the neurogenic and astrogenic yield (ratio of the number of newborn neurons and newborn astrocytes at 30d divided by the number of proliferating neuroprogenitors at 2 h) from both the experimentally estimated data and the Marsaglia simulated data. Experimentally, the newborn neurons were produced from neuroprogenitors at a fairly constant yield across adulthood, although we detected an increase at 6 m but not at 12 m (we ascribe this finding to the larger variability of the 6 m group) (Fig. 3G). Computationally, we found a significantly smaller neuronal yield at 2 m, 6 m, and 12 m compared to the experimentally estimated data, most likely related to the use of the GFAP-locked model, which optimized the simulation of newborn astrocytes. When the NeuN-locked data were used, we found smaller differences between the experimentally estimated and the Marsaglia simulated neuronal yields (Table S11). Importantly, the same trend was found for newborn neurons in both the GFAP-locked and the NeuN-locked models (Table S11), demonstrating a roughly constant neuronal yield from 1 m to 12 m.

In contrast, the astrogenic yield was significantly smaller in 1–2 m than in 6–12 m mice in both the experimentally estimated and the Marsaglia simulated data for the GFAP-locked model (Fig. 3H) and for the NeuN-locked model (Table S11). As a result, the neuron-to-astrocyte ratio decreased from 4.8 at 1 m to 1.2 at 12 m in the experimental data, and from 4.4 at 1 m to 0.8 at 12 m in the Marsaglia simulated data. These data thus demonstrate that newborn neurons and astrocytes are produced with different dynamics across adulthood. In young mice (1–2 m), the proliferating neuroprecursors are largely neurogenic, whereas in mature mice (6–12 m) they produce a similar yield of astrocytes and neurons.

### The increased astrogenic yield across adulthood is associated with the increased BrdU^+^ cell survival during the late critical periods

We finally explored the cellular mechanism underlying the differential yield of neurons and astrocytes. Two observations caught our attention: 1, the astrogenic yield in mature mice was larger compared to young mice (Fig. 3H); and 2, the net survival (the difference between proliferation and apoptosis) of BrdU^+^ newborn cells at 30d in 6–12m mice was higher compared to 1–2 m mice (Fig. 2B,C). These data led us to hypothesize that the increased BrdU + newborn cell net survival at 30d in mature mice (6, 12 m) could be due to the increased production or survival of astrocytes. To test this hypothesis, we created different computational scenarios in which we compared the effect of adding more newborn neurons and/or astrocytes to our basic model described above. To analyze which scenario best matched the experimentally estimated data and the Marsaglia simulation, we utilized the neuron-to-astrocyte ratio, calculated as the ratio between the neuronal yield and the astrocytic yield (Fig. 3G,H).

**Figure 4:**
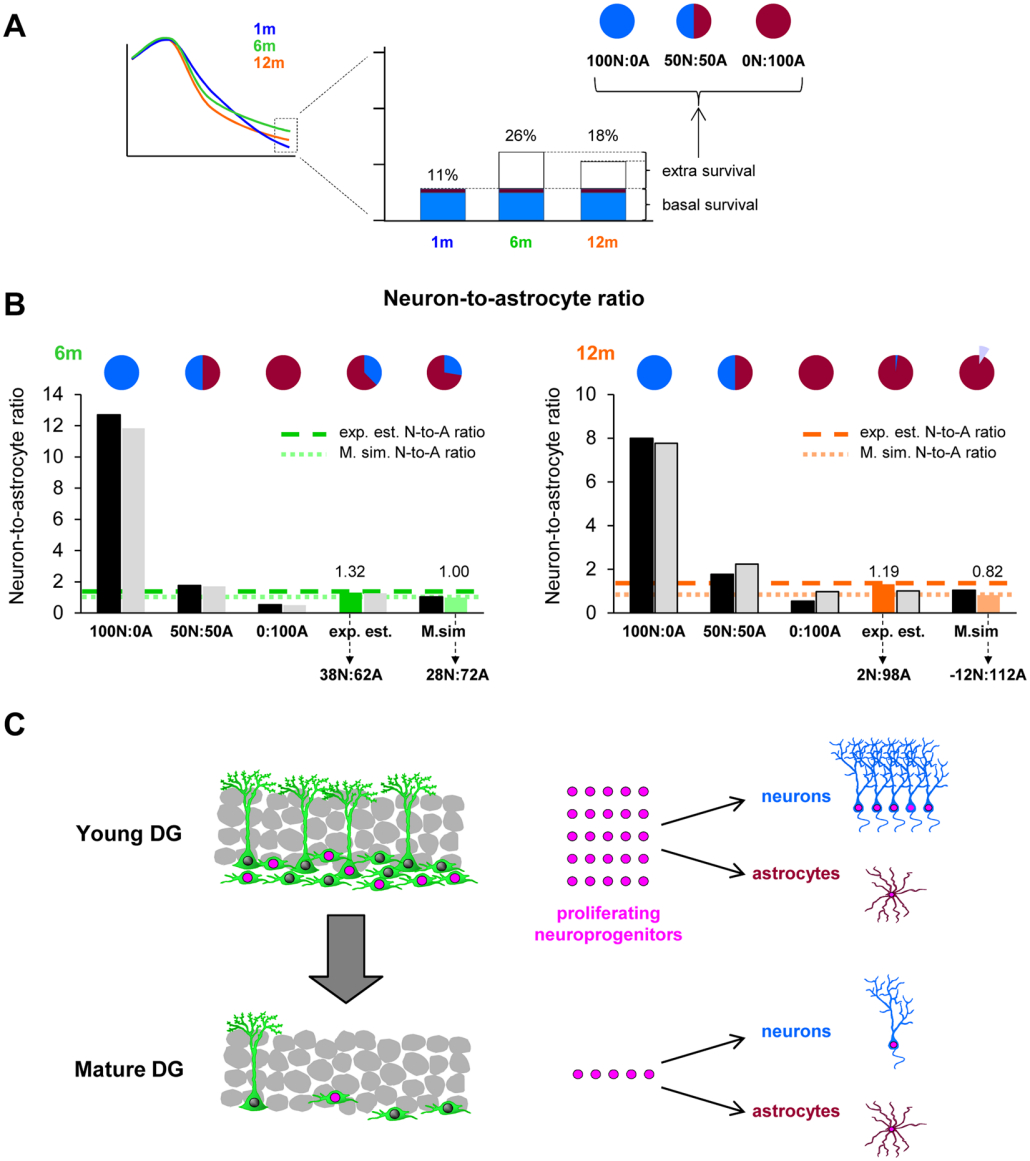
Hippocampal neuroprogenitors produce differential neurogenic and astrogenic outputs throughout adulthood. (**A**) Cartoon represents the strategy used to create different scenarios of newborn neuronal and astrocyte survival (100:0, 50:50, and 0:100%) to account for the increased (extra-) survival found at 6 m and 12 m compared to 1 m. (**B**) Neuron-to-astrocyte ratio from experimentally estimated and the Marsaglia simulated data in 6 and 12 m mice was calculated for each of the scenarios of neuronal and astrocyte survival (100:0, 50:50, and 0:100% of the extra survival). Optimal proportions that allowed reaching the target ratios for the experimentally estimated and the Marsaglia simulated data are drawn on the top. At 12 m, the target ratio from the Marsaglia simulated data could only be reached with negative contributions of neurons (represented by a light purple pie slice). Further data is shown in Table S12. (**C**) Proposed model to explain the major effect of age on the neuroprogenitor population and newborn neuronal and astrocytic yields. The number of cells and dots shown are roughly proportional to the data from 1 m and 12 m mice (young and mature DG, respectively) shown in Figs 1 and 2.

We first modeled the 30d net survival at 6 m and 12 m as a sum of the baseline net survival and neuron/astrocyte production (as at 1 m) and the extra net survival at 6 m and 12 m. This extra survival was modeled with three different contributions of neurons and astrocytes: 100:0, 50:50, and 0:100%, respectively (Fig. 4A). We next examined which scenario best matched the neuron-to-astrocyte ratio (obtained from Fig. 3G,H). The 100N:0A scenario rendered far-off ratios at 6 m and 12 m both in the experimentally estimated and the Marsaglia simulated data (Fig. 4B), demonstrating that neurons were not the main cell type driving the increased net survival at 30d in mature mice compared to young mice.

A better fit of the neuron-to-astrocyte ratio resulted from 50 N:50 A and 0 N:100 A scenarios (Fig. 4B). To determine the optimal scenario of newborn neuron vs newborn astrocyte contribution that rendered neuron-to-astrocyte ratios identical to the experimentally estimated and the Marsaglia simulation, we used a simple iterative parameter search strategy. This strategy was based on comparing two scenarios (initially, 50 N:50 A and 0 N:100 A), determining a middle point in between (i.e.; 25 N:75 A), and comparing this third point with the best fitting of the initial pair (see more details in Supplementary Methods). This procedure was performed iteratively until the goal neuron-to-astrocyte ratio was reached. Using this method, we obtained that at 6 m, the neuron-to-astrocyte ratio was 38 N:62 A (experimentally estimated) and 28 N:72 A (Marsaglia simulation). The relative contribution of neurons to the increased net survival was even smaller at 12 m, as the neuron-to-astrocyte ratio was 2 N:98 A in the experimentally estimated data. The 12 m Marsaglia simulated neuron-to-astrocyte yield could only be optimized when the baseline production of neurons was below the levels of 1 m mice, resulting in proportions of -12N:112 A. The negative proportion of neurons could either be the result of data variability (and refer in fact to an optimal scenario of 0 N:100 A) or represent a reduced production of neurons in favor of astrocytes. Overall, these data demonstrate that the increased net survival of total BrdU^+^ cells during the late critical periods reflects the increased production of astrocytes at 12 m compared to 1 m mice.

## Discussion

Here, we have performed a systematic analysis of the dynamics of the neurogenic cascade both longitudinally (along the cell life, from 2 h to 30d) and transversally (across adulthood, from 1 m to 12 m), using BrdU labeling to quantify proliferating neuroprogenitors and their progeny (i.e., neurons and astrocytes). From the parameters experimentally determined in each age group and for each time point with minimally biologically-imposed restrictions, we generated a simple but useful simulation model of newborn cell dynamics across adulthood to demonstrate age-related changes in newborn cells that have not been evident to date. We report four main findings: 1, the longitudinal dynamics of newborn cell production and survival is largely identical throughout adulthood; 2, the neurogenic yield of proliferating neuroprogenitors is constant over time: the number of newborn neurons is proportional to the number of proliferating neuroprogenitors from 1 m to 12 m; 3, the astrocytic yield of proliferating neuroprogenitor increases in mature mice and is associated with an increased BrdU^+^ cell net survival; and 4, as a result, the niche switches from neurogenic to neuro/astrogenic in mature mice.

First, we analyzed the longitudinal dynamics of newborn cells and found that it was largely similar in young and mature mice, depending only on the initial number of proliferating neuroprogenitors, labeled with BrdU. A similar newborn cell longitudinal decay was obtained in juvenile (1 m) mice using three different BrdU protocols (1x, 4x, and 8x), disregarding any potential BrdU dilution that could hinder the interpretation of our results. Nonetheless, one potential shortcoming of using BrdU to label proliferating neuroprogenitors is that the number of labeled cells depends on blood brain barrier (BBB) permeability. Importantly, hippocampal BBB has been reported to be stable in mice up to 15 m of age^34^, eliminating the possibility that more BrdU enters the brain with advanced age. Thus, the initial labeling of neuroprogenitors relies on three different factors: 1, the relative proportion of different neuroprogenitor subpopulations; 2, their relative mitotic activity; and 3, the relative duration of their cell cycle, particularly of the S phase. The neuroprogenitors labeled in the BrdU protocol used here are mostly TAPs, as the NSCs are a quiescent population of which only a small fraction is mitotically active^14^. Recently, it has become evident that NSCs are very heterogeneous, currently including both radial and horizontal cells^14,15,35^. Radial NSCs are lost over time^14^, potentially diminishing their proliferative capacity along the way^36^. Combined, these two observations (deforestation vs senescence)^17^ have been proposed to explain the well-described phenomenon of decay in neurogenesis during aging^1,5^. The labeling protocol used here does not allow us to discriminate between these hypotheses, as we have not analyzed TAPs, radial, and horizontal NSCs separately. Nonetheless, the combined population of neuroprogenitors behaved homogenously, as they transversally decayed at a constant rate. These data suggest that any changes in one subpopulation may be compensated by opposite changes in another: for example, an increased cell cycle in radial NSCs may be compensated by increased number of TAPs with shorter cell cycles, a hypothesis that should be experimentally addressed.

We next analyzed the transversal decay of proliferating neuroprogenitors and newborn neurons from 1 m to 12 m, and found that they followed an exponential decay with a half-life of 53 and 49d, respectively. These data are in agreement with the decay of the population of proliferating cells (labeled with Ki67) and the population of neuroblasts (labeled with doublecortin) found in a previous study in mice up to 9 m (44 and 53d, respectively)^37^. The age-related decline of neurogenesis is well described in the hippocampus^1–4,14,35–44^), while it does not occur to the same extent but at a lower rate in the other main neurogenic region, the subventricular zone (SVZ)^45–50^. In the hippocampus of many mammalian species, neurogenesis exponentially declines with age, with the sharpest decline associated with sexual maturity, particularly in long-lived animals such as primates^51^. Indeed, we find that in adults the maximum newborn cell decay occurs soon after adolescence both in mice and in humans, with respective half-lives of 1.7 m and 25 y.

Furthermore, the similarity in the transversal decays of the proliferating neuroprogenitor and newborn neuron population led us to propose that both cell types correlated throughout adulthood. Because until now it has not been possible to trace in real time the offspring of neuroprogenitors, we here have developed a computational model of the cascade that allowed us to longitudinally trace cohorts of proliferating cells. Using this model, we have been able to demonstrate that the well-known decrease of newborn neurons with age^1,5^ is largely due to a decreased number of proliferating neuroprogenitors and not compensated by changes in their survival or differentiation rates. One of the principles of neurogenesis postulated by Gerd Kemperman is that “neurogenesis is not regulated by age”^52^ because the largest decay of newborn neurons occurs very early (before 2 y in the human hippocampus)^5^. We here demonstrate another interpretation of this principle, as in fact the rate of production of newborn neurons is independent of age and relies only on the decreased number of proliferating neuroprogenitors.

While newborn neurons decay very fast throughout adulthood (half-life 49d), the newborn astrocyte population decays at a slower rate (half-life 161d). As a result of the slowly decaying addition of newborn astrocytes, the SGZ niche switches from largely neurogenic to similarly neuro-astrogenic. Surprisingly, the production of newborn astrocytes in the hippocampal niche has received little or no attention^6^. Nonetheless, genetic lineage tracing has shown that astrocytes are directly produced from radial NSCs^14,15^ in physiological conditions, possibly from an intermediate, bushy radial NSC (beta subtype)^53^. The contribution of horizontal NSCs to astrocyte generation is not well-established^35^. Different proportions of these two NSC types or differences in their rate of proliferation and differentiation may account for the increased astrocyte yield we found. Alternatively, it is possible that the survival of newborn astrocytes increases in mature mice. These different scenarios should be tested experimentally to understand the cellular and molecular mechanisms underlying the neuro-astrogenic switch undergone by the NSCs with increased age.

Finally, computational models have been proposed before to study different properties of the hippocampal neuro-astrogenic cascade. Ground-breaking work by Aimone *et al*., used mathematical modeling to demonstrate the effect of newborn cell addition to the hippocampal network and its impact on memory encoding, particularly pattern separation^54^. The age-related decay of radial NSCs and TAPs was analyzed by Encinas *et al*., using exponential polynomial functions, leading to the conclusion that radial NSCs decay fast in young animals but their loss slows down as the mice age. In contrast, TAPs decay more slowly at a constant rate, resulting in higher TAP/radial NSCs in older animals^14^. More recently, two complementary approaches have been developed to analyze the cellular dynamics of NSCs. Ziebell *et al*., used ordinary differential equations to model five cellular compartments: NSCs, TAPs, neuroblasts, mature neurons, and astrocytes, each with different rates of self renewal, proliferation, apoptosis, and differentiation^33^. This computational model concluded that an increased proliferation rate of stem cells depletes the stem cell pool and leads to increased number of astrocytes. These predictions were later demonstrated in a mouse model of epilepsy^16^. The predicted increased numbers of astrocytes matches to some extent the findings shown here in physiological aging, although these studies did not analyze the relative neuronal and astrocytic yields. A more complex computational model by Li *et al*., used multiple Bellman-Harris branching and shifted gamma distributions to analyze the cell cycle duration and transit times between NSCs, TAPs, neuroblasts, mature neurons, and apoptotic cells, concluding that apoptosis is a major factor driving newborn cell dynamics^32^. In contrast to the models above, the simulation model presented here does not require analysis of neuroprogenitors behavior (self-renewal, proliferation, apoptosis rates), but rather simplifies the analysis by adopting an “end-point” approach in which 1, all neuroprogenitor populations are analyzed together and 2, proliferation, apoptosis and differentiation are combined together in a “black box” of net survival. This approach works very well for analysis of newborn neurons from 1 m to 12 m, as indeed their production is largely predicted by the number of proliferating neuroprogenitors, without any significant compensatory effects in proliferation or cell survival. In contrast, our simulation model poorly predicts the generation of astrocytes from proliferating neuroprogenitors from 1 m to 12 m, suggesting a yet unknown factor driving the production of astrocytes in the mature hippocampus.

In conclusion, we here provide the stem cell community with a novel and simple simulation model to analyze lineage interactions and make predictions that could be tested later on at the experimental level. Overall, our data show that the hippocampal cascade shifts towards a neuro-astrogenic phenotype with maturity. In physiological conditions, astrocytes have major roles in regulating neuronal communication in tripartite synapses^55^ and in metabolic buffering^56^. Further, preexisting or newborn astrocytes become reactive in a large number of brain diseases, to which they contribute by exacerbating inflammation, compromising BBB function, or releasing neurotoxic compounds^16,57^. Thus, the addition of the slowly decaying newborn astrocytes in the dentate gyrus throughout adulthood may lead to changes of the hippocampal physiology and pathology in the aging hippocampus, to be examined in years to come.

## Electronic supplementary material

Supplementary Information
