# Supplementary material for: A simulation model of neuroprogenitor proliferation dynamics predicts age-related loss of hippocampal neurogenesis but not astrogenesis
Source: Sci Rep. 2017 Nov 28;7:16528. doi: 10.1038/s41598-017-16466-3 (PMC5705784; doi:10.1038/s41598-017-16466-3)
Supplement: Supplementary file 1 — Supplementary Information [file 41598_2017_16466_MOESM1_ESM.pdf]

TITLE:

A simulation model of neuroprogenitor proliferation dynamics predicts age-related loss of hippocampal neurogenesis but not astrogenesis

AUTHORS:

Sol Beccari[1,2,*], Jorge Valero[1,3,*], Mirjana Maletic-Savatic[4], Amanda Sierra[1,2,3]

[1], Achucarro Basque Center for Neuroscience, Leioa, Bizkaia, Spain

[2], University of the Basque Country UPV/EHU, Leioa, Bizkaia, Spain

[3], Ikerbasque Foundation, Bilbao, Bizkaia, Spain

[4], Jan and Dan Duncan Neurological Research Institute at Texas Children's Hospital, Baylor College of Medicine, Houston, TX, USA

SUPPLEMENTARY TEXT

Includes Supplementary Methods, Tables, Figures, and Figure Legends.

SUPPLEMENTARY METHODS

Marsaglia simulation model. The Marsaglia polar method (Marsaglia and Bray, 1964) was implemented to generate pseudorandom and normally distributed populations of $BrdU^+$ cells and their progeny with mean and standard deviation corresponding to those experimentally determined in each age group. Each simulated cell value (v) was generated using the following equation:

$$\boldsymbol{v = mean + (std.dev\ \times u_1\ \times \sqrt{\frac{-2 \times Ln(s)}{s}})}$$

Where mean and std.dev are the experimentally estimated mean and standard deviation of each age group; and u_1 is a random number between -1 and 1 that satisfies the condition:

$$\boldsymbol{s = u_1{}^2 + u_2{}^2} < 1$$

Where u_2 is a random number between -1 and 1 that is paired to u1 to satisfy the condition s < 1 [1].

We first generated a population of 1,000 simulated $BrdU^+$ cells at 2h. For each simulated $BrdU^+$ cell, we generated their progeny at 2, 4, 10, and 30d using the Marsaglia polar method with the following biological-related restrictions based on our observations (**Fig. 1, 2**):

$$\boldsymbol{2h \le 2d \ge 4d \ge 10d \ge 30 \ge (NeuN^+ + GFAP^+\ )}$$

Thus, each simulated $BrdU^+$ cell at 2h originated a string of linked simulated neurons and astrocytes up to 30d.

Two possible strategies were compared to satisfy that ($NeuN^+$ + $GFAP^+$) ≤ 30d: randomly determine percentage of $GFAP^+$ cells and condition the proportion of $NeuN^+$ to this value (GFAP-locked), or viceversa (NeuN-locked). In this supplementary text we summarize the simulated NeuN-locked model (**Table S9**) and the main findings obtained with it: correlation between $NeuN^+$ and $GFAP^+$ at 30d with $BrdU^+$ at 2h (**Table S10**); neuronal and astrocytic yield (**Table S11**); and neuronal and astrocytic contribution to the increased 30d survival in 6 and 12m mice (**Table S12**). The same trends and conclusions were reached using the GFAP-locked strategy reported in the main text and the NeuN-locked strategy shown here.

Data modeling. Curve fitting was used to model the longitudinal and transversal decay of $BrdU^+$, $NeuN^+$, $GFAP^+$, and human Dcx^+ cells. Data modeling was implemented

using GraphPad Prism 5 (GraphPad Software, Incl, San Diego, CA) and optimal curve fitting was determined by Akaike's information criteria (AICc) [2]. We compared six alternative fitting curves: straight, semiLog, Log-Log, exponential decay, exponential decay with plateau, and second order polynomial.

Optimal fitting for all data modeled was obtained with exponential curves (with or without plateau). The only exception was human Dcx data, which fit better with semiLog curves. However, we chose to model human Dcx data with exponential curves to compare the decay and half-life with those obtained with our own data.

Each exponential decay curve was defined by the following formula:

$$\boldsymbol{Y} = (\boldsymbol{Y0} - \boldsymbol{Plateau}) \times \boldsymbol{e}^{(-\boldsymbol{K}\times\boldsymbol{X})} + \boldsymbol{Plateau}$$

Where K is the exponential decay constant and the half-life was calculated as:

$$\boldsymbol{Half - life} = \frac{\boldsymbol{Ln(2)}}{\boldsymbol{K}}$$

Neuronal:Astrocytic contribution to increased survival at 6 and 12m. To understand the survival of $BrdU^+$ cells (neurons + astrocytes) at 30d in 6m and 12m mice, we compared it with the basal survival at 1m. Thus, the number of $BrdU^+$ cells at 6m and 12m was modeled as the basal number of cells produced (using the net survival and differentiation rates as in 1m) and a number of extra cells that could be calculated as the sum of extra neurons and extra astrocytes in different proportions. We explored different scenarios with different neuronal and astrocyte contributions. In addition, we performed an iterative algorithm to search for optimal contributions of neurons and astrocytes. We finally tested which scenario fit best with the experimentally determined and the Marsaglia simulation data comparing the neuron-to-astrocyte (N-to-A) ratios.

<u>1. Basal number of neurons and astrocytes at 30d in 6 and 12m mice.</u>

The basal number of neurons and astrocytes (basal N and basal A, respectively) at 6 and 12m was calculated using the basal survival (1m % survival) and differentiation rates (1m % NeuN and 1m % GFAP, respectively):

$$\boldsymbol{basal\ N_{6/12m}} = \boldsymbol{BrdU2h_{6/12m}} \times \frac{\%\ \boldsymbol{survival_{1m}}}{\boldsymbol{100}} \times \frac{\%\ \boldsymbol{NeuN_{1m}}}{\boldsymbol{100}}$$

$$\boldsymbol{basal\ A_{6/12m}} = \boldsymbol{BrdU2h_{6/12m}} \times \frac{\%\ \boldsymbol{survival_{1m}}}{\boldsymbol{100}} \times \frac{\%\ \boldsymbol{GFAP_{1m}}}{\boldsymbol{100}}$$

2. Extra number of neurons and astrocytes at 30d in 6 and 12m mice.

The extra number of neurons and astrocytes (extra N and extra A, respectively) at 6 and 12m was calculated using the extra survival and extra differentiation rates (extra % NeuN and % GFAP, respectively):

$$\boldsymbol{extra\ N_{6/12m}} = \boldsymbol{BrdU2h_{6/12m}} \times \frac{\%\ \boldsymbol{extra\ survival_{6/12m}}}{\mathbf{100}} \times \frac{\%\ \boldsymbol{extra\ NeuN_{6/12m}}}{\mathbf{100}}$$

$$\boldsymbol{extra\ A_{6/12m}} = \boldsymbol{BrdU2h_{6/12m}} \times \frac{\%\ \boldsymbol{extra\ survival_{6/12m}}}{\mathbf{100}} \times \frac{\%\ \boldsymbol{extra\ GFAP_{6/12m}}}{\mathbf{100}}$$

Where the extra survival was calculated as a difference with the 1m % survival:

$$\%\ \boldsymbol{extra\ survival_{6/12m}} = \%\ \boldsymbol{survival_{6/12m}} - \%\ \boldsymbol{survival_{1m}}$$

The extra differentiation rates were tested in different proportions (100N:0A, 50N:50A, and 0N:100A). The total number of neurons and astrocytes were calculated as the sum of basal + extra cells in each scenario. The N-to-A ratios were calculated and compared to the target ratios (obtained in experimentally estimated and Marsaglia simulation data) (**Fig. 4B**).

3. Optimization of neuronal and astrocyte contributions.

We used a simple iterative parameter search algorithm to find the combination of neuronal and astrocytic contribution to the extra survival that resulted in the N-to-A ratio closest to the target ratios. The optimal neuron and astrocyte contributions were determined independently for the experimentally estimated and the Marsaglia simulation data. Initially, the algorithm tested two different combinations of neuronal and astrocyte contributions (C1=100N:0A and C2=0N:100A) and selected the optimal combination that rendered the smallest absolute difference to target N-to-A ratio. In the next step, the selected combination was compared with a newer combination calculated as:

$$C3 = optimal\ C\ \pm \frac{|C2 - C1|}{2}$$

Differences with opposite signs indicate that the optimal combination was located between the previous two combinations. In this case, the term $\frac{|C2-C1|}{2}$ was added to C1 or subtracted from C2 depending on which one was selected as optimal, respectively. In contrast, differences with identical signs indicate that the optimal combination was either below C1 or above C2. In this latter case, the term $\frac{|C2-C1|}{2}$ was added to C2 or

subtracted from C1 depending on which one was selected as optimal, respectively. The algorithm was performed iteratively until the N-to-A ratios obtained reach the minimal possible absolute difference (0.001) with those obtained using experimentally estimated or random Marsaglia simulation data.

SUPPLEMENTARY TABLES

Table S1. Raw data of BrdU$^+$ cells in the hippocampus. Total number of BrdU$^+$ cells in the hippocampus at 1m, 2m, 6m, and 12m, from 2h to 30d after the BrdU injection using a 4x BrdU paradigm. Each value was obtained from a different animal.

Days	1m	2m	6m	12m
**2h**	2850	1873	258	90
	2480	1680	270	144
	2862	1548	270	186
	2340	2067	312	96
	2380	1800		126
				132
				90
**2d**	3060	2267	342	150
	2793	2094	360	192
	2973	2407	336	168
	3173	2130	300	132
	3307	2427		120
				138
				132
**4d**	1593	1293	162	54
	1593	1580	165	84
	1718	1133	173	60
	2130	1500	144	60
	1740	1233		108
				48
				42
**10d**	985	720	120	60
	1040	804	155	66
	870	473	128	24
	1050	473	108	42
	876	467		30
				66
				54
**30d**	333	240	48	24
	240	186	72	26
	342	127	60	22
	204	207	108	20
	260	156		24
				22
				20
				20
				20
				30

Table S2. Raw data of % $BrdU^+$ cells expressing NeuN in the hippocampus. % of $NeuN^+$, $BrdU^+$ cells over total $BrdU^+$ cells at 1m, 2m, 6m, and 12m, 30d after the BrdU injection. Each value was obtained from a different animal.

Age	%NeuN
**1m**	87.3
	87.9
	81.1
	93.7
**2m**	79.4
	75.7
	75.6
	86.9
**6m**	71.1
	76.9
	42.9
	60.0
	50.0
	37.5
**12m**	37.5
	66.7
	41.2
	57.9
	50.0

Table S3. Raw data of % BrdU$^+$ cells expressing GFAP in the hippocampus. % of GFAP$^+$, BrdU$^+$ cells over total BrdU$^+$ cells at 1m, 2m, 6m, and 12m, 30d after the BrdU injection. Each value was obtained from a different animal.

Age	%GFAP
**1m**	15.4
	10.3
	12.9
	10.5
	13.6
	24.0
	23.3
	25.7
	27.5
**2m**	6.3
	6.7
	29.4
	16.7
	31.0
	12.8
	16.1
	22.0
	28.1
**6m**	33.3
	33.3
	25.0
	50.0
	30.8
	40.0
	61.5
	46.2
	45.5
	62.5
**12m**	33.3
	25.0
	100.0
	75.0
	85.7
	0.0
	0.0
	33.3
	100.0
	100.0
	37.5
	0.0
	22.2
	40.0
	11.0
	50.0
	40.0
	28.6
	33.3
	36.4

Table S4. Raw data of % BrdU$^+$ cells expressing Ki67 in the hippocampus. % of Ki67$^+$, BrdU$^+$ cells over total BrdU$^+$ cells at 1m, 2m, 6m, and 12m, 2d after the BrdU injection. Each value was obtained from a different animal.

Age	% Ki67
**1m**	25.9
	16.8
	31.3
	22.1
	28.8
	31.5
	30.4
	31.4
	17.2
	27.8
**2m**	18.6
	14.6
	32.5
	23.8
	29.2
	30.1
	26.2
	31.9
	20.7
	24.6
**6m**	26.9
	38.5
	28.6
	31.8
	41.7
	21.6
	32.5
	25.6
**12m**	50.0
	25.0
	30.0
	20.0
	20.0
	20.0
	23.1
	33.3
	33.3
	37.5
	25.0
	44.4
	25.0
	20.0

Table S5. Raw data of BrdU$^+$ cells in the hippocampus (BrdU 1x and 8x). Total number of BrdU+ cells in the hippocampus at 1m, 2m, 6m, and 12m, from 2h to 30d after the BrdU injection using 1x and 8x BrdU paradigm. Data originally published in [3]. Each value was obtained from a different animal.

Days	1x	8x
**2d**	53180	162440
	57955	104464
	58770	138043
		103162
**3d**	47709	
	49137	
	40457	
**4d**	35931	90764
	26769	93978
	27224	75699
		86170
**6d**		105994
		84468
		60916
**8d**	18491	41509
	25115	25418
	35320	46730
	40763	
**11d**	21418	20848
	21914	26623
	26373	20916
**15d**	15651	21415
	14638	14927
	8752	11659
	12324	
**18d**	14052	19030
	7080	15507
	14280	20828
		17696
**22d**	6994	11728
	8454	10274
	6409	7474
	6936	
**32d**	12736	13285
	7014	19750
	7525	8729

Table S6. Experimentally estimated and Marsaglia simulated data. Comparison between the experimentally estimated and Marsaglia simulated populations of $BrdU^+$, $NeuN^+$, and $GFAP^+$ cells along the time course and across adulthood. For each population, the mean number of cells, the standard deviation (in parenthesis) and the sample size (N) are indicated.

**Age**	**Cell population**	**Estimated cells**	**Simulated cells**
**1m**	BrdU (2h)	2582 (255) N=5	2592 (254) N=1000
	BrdU (2d)	3061 (195) N=5	3087 (187) N=1000
	BrdU (4d)	1755 (221) N=5	1747 (221) N=1000
	BrdU (10d)	964 (87) N=5	968 (88) N=1000
	BrdU (30d)	276 (60) N=5	275 (59) N=1000
	NeuN (30d)	241 (47) N=4	216 (50) N=1000
	GFAP (30d)	50 (15) N=9	50 (22) N=1000
**2m**	BrdU (2h)	1794 (196) N=5	1790 (203) N=1000
	BrdU (2d)	2265 (153) N=5	2270 (147) N=1000
	BrdU (4d)	1348 (187) N=5	1347 (190) N=1000
	BrdU (10d)	588 (162) N=5	585 (158) N=1000
	BrdU (30d)	183 (44) N=5	181 (43) N=1000
	NeuN (30d)	145 (31) N=4	134 (34) N=1000
	GFAP (30d)	34 (11) N=9	36 (18) N=1000
**6m**	BrdU (2h)	278 (24) N=4	278 (24) N=1000
	BrdU (2d)	335 (25) N=4	336 (24) N=1000
	BrdU (4d)	161 (12) N=4	161 (12) N=1000
	BrdU (10d)	128 (20) N=4	125 (17) N=1000
	BrdU (30d)	72 (26) N=4	69 (24) N=1000
	NeuN (30d)	41 (18) N=6	30 (14) N=1000
	GFAP (30d)	31(18) N=10	30 (14) N=1000
**12m**	BrdU (2h)	123 (35) N=7	125 (35) N=1000
	BrdU (2d)	147 (25) N=7	161 (25) N=1000
	BrdU (4d)	65 (23) N=7	64 (23) N=1000
	BrdU (10d)	49 (17) N=7	40 (17) N=1000
	BrdU (30d)	23 (4) N=10	21 (6) N=1000
	NeuN (30d)	11 (1) N=5	8 (4) N=1000
	GFAP (30d)	9 (1) N=20	10 (6) N=1000

Table S7. Parameters for longitudinal and transversal newborn cell decay fitting. The table describes the optimal fitting equation shown in each corresponding figure; the cell population that it relates to; the equation parameters (Y0 in days, except in *, in years; the survival plateau in %; the K value); and the coefficient of determination, R^2. For each parameter, the estimated value and standard deviation of the estimate (in parenthesis) are indicated. Data for **Fig. S2E** was originally published in [3] and for **Fig. S2F, G** in [4].

Referring Fig.	Fitting Equation	Group	Y0 (d)	Plateau (%)	K	R^2
**2C**	Y=(Y0 - Plateau)*exp(-K*X) + Plateau	1m BrdU	152.0 (12.5)	12.8 (3.3)	0.251 (0.037)	0.956
		2m BrdU	157.0 (11.8)	10.3 (3.0)	0.255 (0.033)	0.966
		6m BrdU	285.1 (71.5)	29.4 (3.3)	0.644 (0.136)	0.928
		12m BrdU	282.3 (71.3)	22.1 (3.4)	0.605 (0.131)	0.850
**2G**	Y=Y0*exp(-K*X)	Sim BrdU (2h)	149.7 (7.9)	-	0.396 (0.035)	0.978
		Sim NeuN (30d)	149.5 (9.5)	-	0.429 (0.043)	0.970
		Sim GFAP (30d)	104.8 (6.3)	-	0.129 (0.018)	0.854
**3D**	Y=(Y0 - Plateau)*exp(-K*X) + Plateau	Sim 4x BrdU (1m)	154.0 (1.0)	13.0 (0.2)	0.259 (0.003)	0.951
**S2E**	Y=(Y0 - Plateau)*exp(-K*X) + Plateau	1x BrdU (1m)	116.6 (10.5)	12.4 (5.5)	0.141 (0.033)	0.869
		8x BrdU (1m)	168.1 (19.2)	11.1 (3.1)	0.294 (0.046)	0.903
**S2F**	Y=Y0*exp(-K*X)	hDCX (<2y)	87.8 (10.6)	-	0.004 (0.001)	0.676
**S2G**	Y=Y0*exp(-K*X)	hDCX (>2y)	166.1 (17.0)*****	-	0.028 (0.005)	0.564

Table S8. Parameters for correlating simulated data. The table describes the correlating equation shown in each corresponding figure; the cell population that it relates to; the equation parameters (a, b, c); the coefficient of determination, R^2; and the p-value (for linear correlations only). For each parameter, the estimated value and standard deviation of the estimate (in parenthesis) are indicated. Parameter a was set to zero to reflect that without $BrdU^+$ cells there would not be any newborn neuron nor astrocyte.

Referring Fig.	Correlation Equation	Population Correlation	a	b	R^2	p value
**3A**	Y=a+b*X	NeuN (30d)/ BrdU (2h)	0.000 (0.000)	0.092 (0.002)	0.981	<0.0001
**3B**	Y=a+b*X	GFAP (30d)/ BrdU (2h)	0.000 (0.000)	0.020 (0.002)	0.444	<0.0001
**3E**	Y=a+b*X	sim NeuN (30d)/ sim BrdU (2h)	0.000 (0.000)	0.080 (0.000)	0.850	<0.0001
**3F**	Y=a+b*X	sim GFAP (30d)/ sim BrdU (2h)	0.000 (0.000)	0.020 (0.000)	0.067	<0.0001

Table S9. NeuN-locked Marsaglia simulated data. Comparison between the experimentally estimated and Marsaglia simulated populations of $NeuN^+$, and $GFAP^+$ cells along the time course and across adulthood in the NeuN-locked model. For each population, the mean number of cells, the standard deviation (in parenthesis), and the sample size are indicated.

Age	Cell population	Estimated cells	Simulated cells NeuN-locked
**1m**	NeuN (30d)	241 (47) N=4	240 (54) N=1000
	GFAP (30d)	50 (15) N=9	24 (14) N=1000
**2m**	NeuN (30d)	145 (31) N=4	144 (36) N=1000
	GFAP (30d)	34 (11) N=9	23(12) N=1000
**6m**	NeuN (30d)	41 (18) N=6	38 (17) N=1000
	GFAP (30d)	31(18) N=10	22 (12) N=1000
**12m**	NeuN (30d)	11 (1) N=5	11 (4) N=1000
	GFAP (30d)	9 (1) N=20	6 (4) N=1000

Table S10. Parameters for correlating simulated data in the NeuN-locked model. The table describes the correlating equation parameters (a, b, c); the coefficient of determination, R^2; and the p-value. For each parameter, the estimated value and standard deviation of the estimate (in parenthesis) are indicated. Parameter a was set to zero to reflect that without $BrdU^+$ cells there would not be any newborn neuron nor astrocyte.

**Correlation Equation**	**Population Correlation**	**a**	**b**	**R^2**	**p value**
Y=a+b*X	sim NeuN (30d)/ sim BrdU (2h)	0.000 (0.000)	0.088 (0.000)	0.840	<0.0001
Y=a+b*X	sim GFAP (30d)/ sim BrdU (2h)	0.000 (0.000)	0.011 (0.000)	-0.297	<0.0001

Table S11. Neuron and astrocyte yields in the NeuN-locked model. Comparison between neuron and astrocyte yields obtained from the experimentally estimated and the simulated populations of $NeuN^+$ and $GFAP^+$ cells using the NeuN-locked model. For each population, the mean yield, the standard deviation (in parenthesis) and the sample size are indicated.

Age	Cell population	Estimated yield	Simulated yield NeuN-locked
**1m**	NeuN	0.09	0.09 (0.02) N=1000
	GFAP	0.02	0.01 (0.00) N=1000
**2m**	NeuN	0.08	0.06 (0.02) N=1000
	GFAP	0.02	0.01(0.01) N=1000
**6m**	NeuN	0.15	0.12 (0.05) N=1000
	GFAP	0.11	0.07 (0.04) N=1000
**12m**	NeuN	0.09	0.07 (0.03) N=1000
	GFAP	0.08	0.04 (0.02) N=1000

Table S12. Neuronal and astrocyte contributions to reach the target Neuron-to-Astrocyte ratios in 6 and 12m mice. Comparison between neuronal and astrocytic contribution to extra survival in 6 and 12m mice to reach the target Neuron-to-Astrocyte ratio obtained from experimentally estimated and Marsaglia simulated (NeuN-locked model) data.

**Age**	**Estimated cells N:A contribution**	**Simulated cells N:A contribution NeuN-locked**
**6m**	38:62	44:56
**12m**	2:98	24:76

SUPPLEMENTARY FIGURES

Supplementary Figure S1

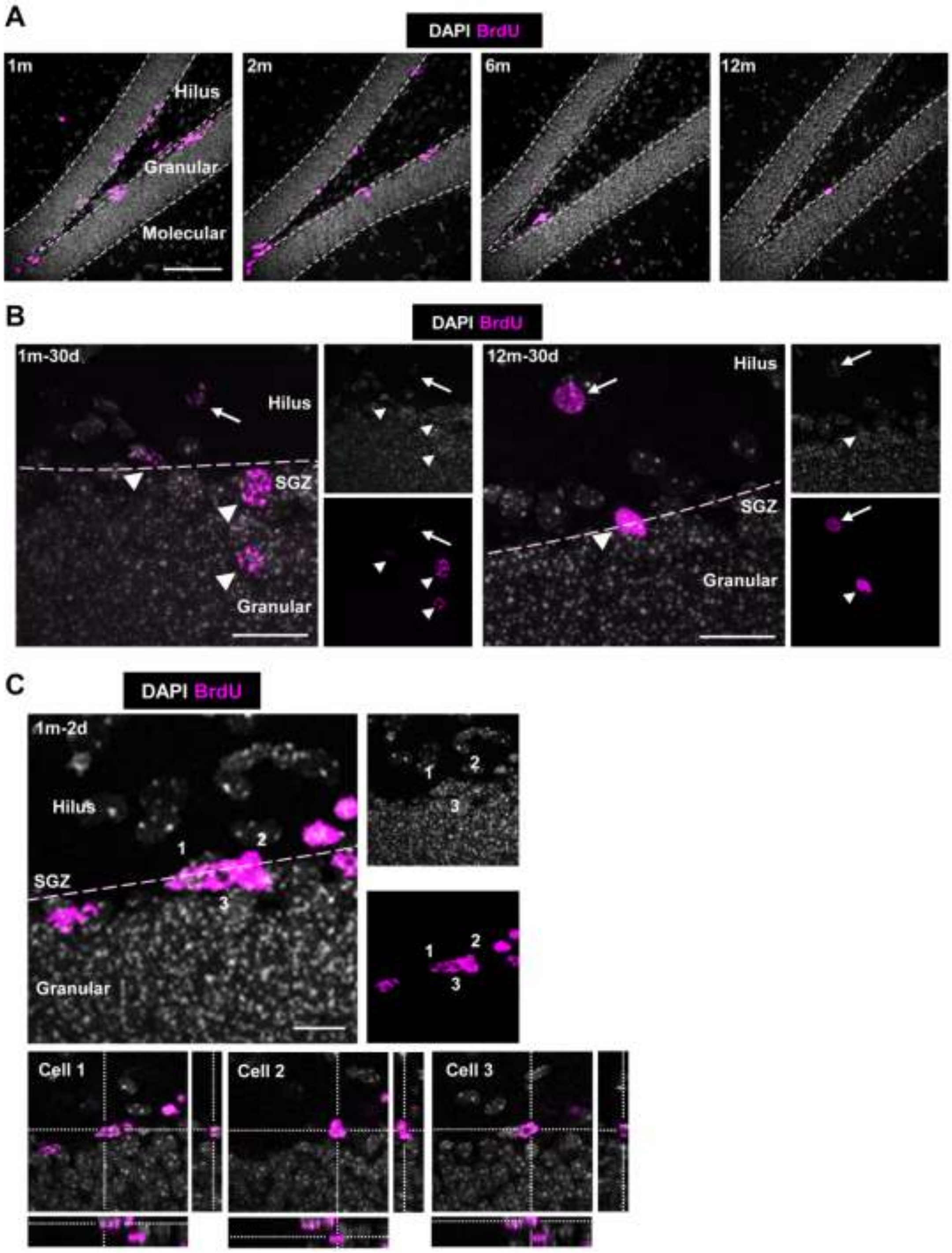

Supplementary Figure S2

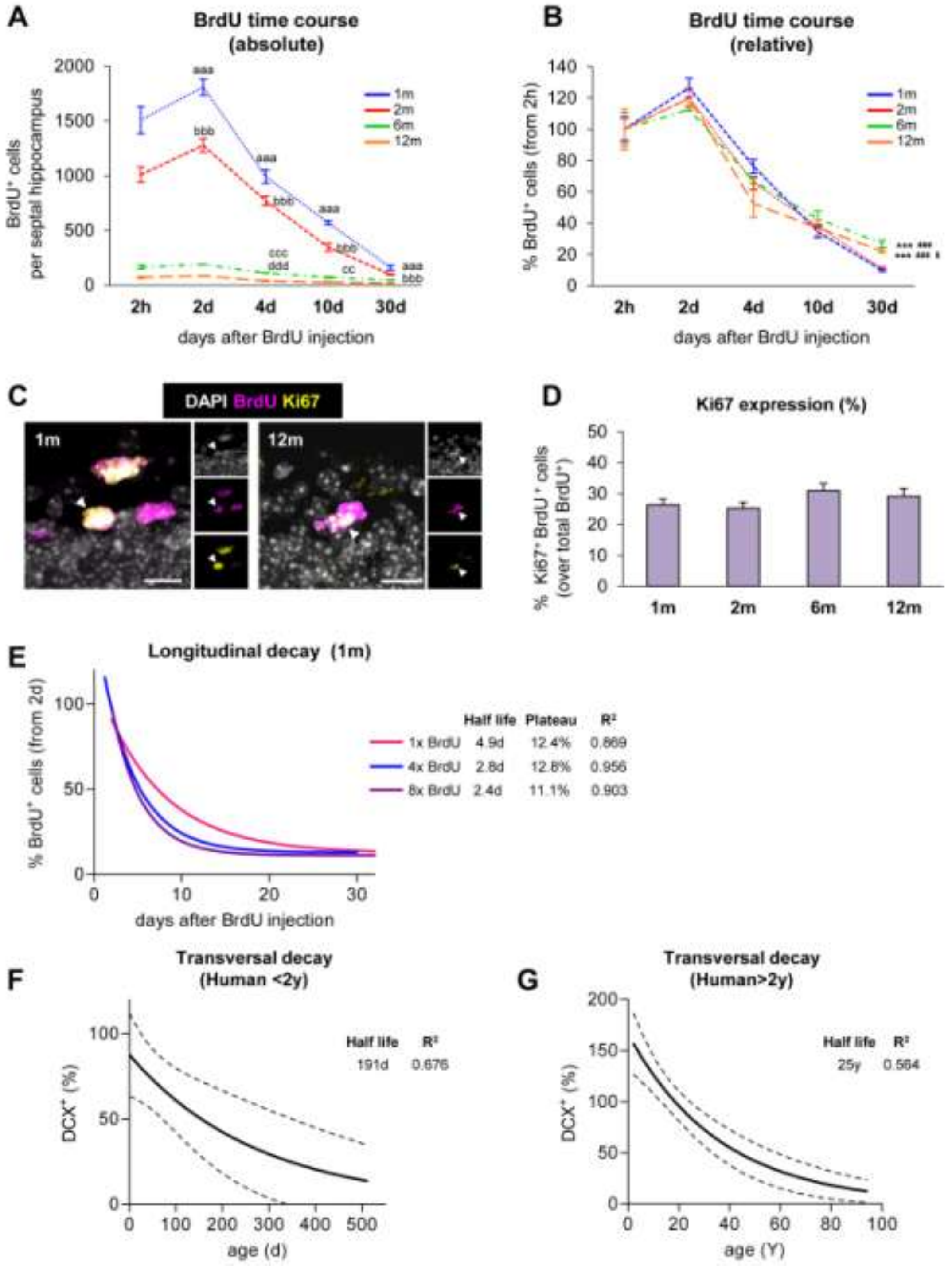

SUPPLEMENTARY FIGURE LEGEND

Figure S1. BrdU counting method.

A, Low magnification confocal image of the dentate gyrus showing the hilus, the granular and the molecular layers, delimited by a pink discontinuous line at 2h postinjection in 1m, 2m, 6m, and 12m mice. Nuclei are shown in white (DAPI) and BrdU cells in magenta. The same images are shown in **Fig. 1A.**

B, High magnification confocal image of $BrdU^+$ cells (magenta) in at 30d postinjection in 1m and 12m mice. DAPI-labeled nuclei are shown in white. Arrowheads point to $BrdU^+$ cells in the SGZ or the granular layer (included in the study); arrows point to $BrdU^+$ cells in the hilus (excluded from the study).

C, High magnification confocal image of a cluster of three $BrdU^+$ cells (magenta) in at 2d postinjection in 1m mice. DAPI-labeled nuclei are shown in white. The orthogonal projection of each cell is shown to indicate that identification of individual cells from clusters requires refocusing through the z plane.

Scale bars = 100µm (**A**), 20µm (**B, C**); z = 16.5µm (A), 16µm (**B**, 1m), 15µm (**B**, 12m), 22µm (**C**)

Figure S2. Age-related decline in hippocampal neurogenesis.

A, Absolute number of $BrdU^+$ cells in the septal hippocampus along the BrdU time course. a, b, c and d represent significance with prior time point for 1m, 2m, 6m and 12m mice, respectively. One symbol is used to represent $p<0.05$, two for $p<0.01$, and three for $p<0.001$. N=5 for 1mo and 2mo mice at 2h, 2d, 4d, 10d, 30d; N=4 for 6mo mice at 2h, 2d, 4d, 10d, 30d; N=7 for 12mo mice at 2h, 2d, 4d and 10d, and N=10 for 12mo mice at 30d.

B, Percentage of $BrdU^+$ cells per septal hippocampus from the number of $BrdU^+$ cells found at 2h. Symbols indicates $p<0.01$ between 2m vs 12m; and *, #, $ represent $p<0.001$ between 1 and 2m vs 6m, 1 and 2m vs 12m, and 6m vs 12m, respectively.

Points represent mean ± SEM. N per group as in **Fig. S2A**.

C, Representative confocal z-stacks of the DG of 1 and 12m mice at 2d after BrdU injection. $BrdU^+$ cells (magenta) were co-labeled with the proliferation marker Ki67 (yellow). DAPI staining indicated cell nuclei (in white). Scale bars = 30µm; z = 18µm.

D, Expression of Ki67 in the $BrdU^+$ cells at 2d (in %). N=10 at 1m, 2m; N=8 at 6m; N=14 at 12m. 1-way ANOVA analysis $F(3,38)=1.113$, $p=0.356$. Bars represent mean ± SEM.

E, Longitudinal decay of 1m simulated $BrdU^+$ cells over the time course (2d-30d), calculated as an exponential curve with plateau obtained with three BrdU

administration protocols: 4x (shown here), and 1x and 8x (from [3]). For each population, the fitting curve, cell half-life, survival plateau (%), and R^2 are shown. N as indicated in **Fig. 1A** (4x BrdU) and **Fig. 3D** (1x Brdu) and (8x BrdU).

F, Transversal decay of Dcx^+ cells in infants under 2y (from [4]). The fitting curve and 95% confidence interval (dotted line), half-life, and R^2 are shown.

G, Transversal decay of Dcx^+ cells in children over 2y and adults (from [4]). The fitting curve and 95% confidence interval (dotted line), half-life, and R^2 are shown.

SUPPLEMENTARY REFERENCES:

1 Marsaglia, G. & Bray, T. A. A Convenient Method for Generating Normal Variables. *SIAM Review* **6**, 260-264 (1964).

2 Burnhan, K. P. & Anderson, D. R. *Model Selection and Multimodel Inference: A Practical Information-Theoretic Approac*. (Springer-Verlag, 2002).

3 Sierra, A. *et al.* Microglia shape adult hippocampal neurogenesis through apoptosis-coupled phagocytosis. *Cell Stem Cell* **7**, 483-495, doi:S1934-5909(10)00437-6 [pii] 10.1016/j.stem.2010.08.014 (2010).

4 Knoth, R. *et al.* Murine features of neurogenesis in the human hippocampus across the lifespan from 0 to 100 years. *PLoS One* **5**, e8809, doi:10.1371/journal.pone.0008809 (2010).
